# Insights Into the Association Between Myasthenia Gravis and Depression: A Clinical Case Study

**DOI:** 10.7759/cureus.43682

**Published:** 2023-08-18

**Authors:** Nicole Ann E Villa, Gina Maria P Fiore, Eduardo D Espiridion

**Affiliations:** 1 Psychiatry, Drexel University College of Medicine, Wyomissing, USA; 2 Psychiatry, Reading Hospital, Tower Health Systems, West Reading, USA; 3 Psychiatry, West Virginia School of Osteopathic Medicine, Lewisburg, USA; 4 Psychiatry, Drexel University College of Medicine, Philadelphia, USA; 5 Psychiatry, Philadelphia College of Osteopathic Medicine, Philadelphia, USA

**Keywords:** disease burden, quality of life, mental health, autoimmune disease, depression, myasthenia gravis

## Abstract

Myasthenia gravis (MG) is a serious and debilitating autoimmune disease characterized by muscle weakness, shortness of breath, and issues affecting the eyes, limbs, throat, and speech. Given the intense physical toll of the disease, it is unsurprising that higher rates of depression are observed among MG patients.

We present a case involving a 30-year-old female patient who was admitted to the hospital for MG exacerbation and had a psychiatric consultation for worsening depression symptoms. The patient acknowledged symptoms of sad mood, crying spells, anhedonia, fatigue, insomnia, and inappropriate guilt. She admits to psychosocial stressors of her declining health, recent job loss, and low self-esteem due to weight gain. Past medical history includes a thymectomy and a total thyroidectomy that caused postsurgical-acquired hypothyroidism. She is currently on prednisone and pyridostigmine for her MG.

The patient has many potential causes of her increased depressive symptoms, including her medications, psychosocial stressors, and her past medical history, in addition to her MG. However, the literature shows higher incidence rates of depression in MG patients compared to both healthy controls and controls with other comparable chronic conditions, as well as shows a positive association between increased depressive symptoms and MG severity. Thus, these findings prompt the consideration of possible physiological interplay between the two diseases and encourage further research into the association between MG and depression.

## Introduction

Myasthenia gravis (MG) is a serious and debilitating autoimmune disease characterized by muscle weakness, most typically causing shortness of breath, as well as affecting the eyes, limbs, throat, and speech [1]. It is most prevalent in females aged around 40 years and males aged around 60 years [1,2]. While some MG patients may be able to function normally and carry out their typical daily activities, serious cases or a myasthenic crisis involve respiratory failure and require ventilation. MG is chronic, and there is no known cure, but treatments include medications like acetylcholinesterase inhibitors aimed at improving acetylcholine reception at nervous and muscular junctures, as well as immunosuppressive drugs and thymectomy [1,3].

Depression is prevalent among patients with MG [4]. This is unsurprising, considering the significant impact the disease frequently has on patients' lives, including their ability to work, engage in physical activities, or even communicate [1,4]. However, despite the physical burdens of the disease, there are higher rates of depression among MG patients when compared to similar conditions like autoimmune diseases such as multiple sclerosis and systemic lupus erythematosus. These conditions, much like MG, can be characterized by progressive muscle weakness [5]. Thus, this report presents the case of a patient experiencing depression in concordance with a myasthenic exacerbation and explores the relationship between the two factors. 

## Case presentation

The patient, a 30-year-old female, was admitted to the medical floor of a community hospital due to MG exacerbation. She has been diagnosed with MG for approximately five years. There was ambulatory dysfunction, which needed assistance for her to walk to the bathroom. She required intravenous immunoglobulin treatment. She was also prescribed prednisone 30 mg daily and pyridostigmine 60 mg five times a day.

A psychiatric consultation was requested because of worsening depression and anxiety symptoms. The patient acknowledged being depressed with symptoms of sad mood, crying spells, anhedonia, fatigue, insomnia, and inappropriate guilt. She also admitted to excessive anxieties and ruminative worries. There were no suicidal or homicidal ideations. There were no manic or psychotic symptoms. She denied any substance use disorder problems. She admitted to psychosocial stressors of her declining health and her job loss as triggers. Her self-esteem decreased too because of her 20-pound weight gain in the past year. She was maintained on her current psychiatric medication regimen, which included mirtazapine, bupropion, and alprazolam. Psychotherapy was felt to be helpful during the psychiatrist visits during this hospitalization. She agreed to continue outpatient psychiatric follow-ups for medication management and psychotherapy.

The patient underwent a thymectomy, which did not show any malignancy. She also had a history of postsurgical-acquired hypothyroidism after a total thyroidectomy for a papillary thyroid cancer. She was on maintenance levothyroxine treatment. She also had multiple therapeutic plasma exchange, with the most recent one being three months before this admission. 

## Discussion

Aside from MG, this patient presented with many other potential causes of her depressive symptoms, including her medications, psychosocial stressors, and her past medical history, which were worth considering.

The most notable medication among the potential causes of depression was prednisone. The patient was taking 30 mg daily, and studies have established a connection between corticosteroid treatment and heightened depressive symptoms [6-8]. Prednisone has been associated with mood changes in patients receiving long-term and short-term steroid treatment. In a prospective study involving patients with inflammatory bowel disease, a mood change rate of 49% was reported after two weeks of undergoing daily prednisone treatments at a dosage of 40 mg. These results did include some depressive symptoms as measured by the Beck Depression Inventory-II but were mostly related to increased hypomania symptoms. The mood changes ceased with the termination of prednisone treatment [6]. A study focusing on the psychiatric effects of prednisone treatment for six months or longer found that long-term prednisone effects may tend toward depression more than mania when compared with the psychiatric effects of short-term treatment. In this study, subjects had either asthma or rheumatic illness, and the patients receiving corticosteroid treatment had significantly higher scores on the Hamilton Rating Scale for Depression compared to controls [7]. Another study of the psychiatric effects of prednisone treatment showed that severe prednisone-dependent asthma patients had significantly higher scores for depressive symptoms on the Hospital Anxiety and Depression Scale (HADS) compared to non-prednisone-dependent asthma patients [8]. Collectively, these findings indicate that depression and mood changes can be adverse side effects of taking prednisone. While none of the studies described involved MG patients, as comparable results were found across three different illnesses all treated with prednisone, this suggests that similar results could occur in MG patients taking prednisone. While the link between prednisone and depression specifically in MG patients is unclear, the literature suggests that the patient’s corticosteroid treatment could be exacerbating her depression.

Psychosocial triggers such as her recent job loss and lowered self-esteem due to recent weight gain may be contributing to the patient’s increased depression. Losing one’s job can place elevated levels of stress on an individual, and the majority of people surveyed in a Dutch 2021 study experienced some form of grief reaction, anxiety, and/or depressive symptoms [9]. Aligned with current findings, the patient’s dissatisfaction with her weight likely contributes to her depression as well. Significant relationships between increased depressive symptoms and dissatisfaction with being overweight, or even just a subjective perception of being overweight, have been observed in adolescents [10], middle-aged women, and older women alike [11]. These psychosocial elements of the patient’s health are considerations for her recent worsening depression.

Associations between thyroid disorders and depression have long been studied, and given the patient’s past thyroid cancer and thyroidectomy, this is also a reasonable consideration for the cause of her depression. However, recent studies, specifically large meta-analyses, are only finding slight associations between hypothyroidism and depression [12] and few instances of statistical significance, despite finding positive associations between hypothyroidism and depression [13,14]. A 2018 study from Korea found hyperthyroidism to be associated with clinical depression, but no association was found for hypothyroidism [15]. While there is no way to rule out hypothyroidism as a contributing factor to the patient’s depression, the literature’s mixed findings concerning its relationship with depression, and the worsening of the patient’s depression coinciding with a myasthenia exacerbation and not with the onset of her hypothyroidism, suggests that other factors may be at play. In addition to a thyroidectomy, this patient also had a thymectomy. However, a study of over 3,000 MG patients found that thymectomy was associated with higher health-related quality of life (HRQoL) scores compared to MG patients with no thymectomy. These results provide data to support that her thymectomy is unlikely to be a contributing factor to her depression as it has not been shown to lower HRQoL scores [2]. 

Despite these potential causes of the patient’s depression, the patient's MG disease itself is a consideration for the exacerbation of her depression. A large survey with over 1,500 MG patients found that women displayed more anxiety and depressive symptoms compared to males, and the study by Marbin et al. with over 3,000 MG patients supported this finding, stating that women had significantly lower HRQoL scores compared to men [2,3]. These results suggest that the patient's female sex may have predisposed her to a diagnosis of depression comorbid with her MG.

MG has been linked to higher HADS scores and the overall incidence of depression compared to the healthy population [3,5], and increased severity of depression and anxiety is associated with higher severity of MG. MG also has higher rates of depression compared to patients with other chronic conditions such as multiple sclerosis, lupus, dementia, and ALS [5,16-19]. The higher incidence rates of depression in MG patients compared to both healthy controls and controls with other comparable chronic conditions, along with the positive association between increased depressive symptoms and MG severity, collectively raise the question of potential physiological interactions between the two conditions.. 

## Conclusions

This case report examined a patient with depression comorbid with her MG exacerbation. She had several possible contributing factors to her depression, including corticosteroid medication, psychosocial stressors such as job loss and dissatisfaction with her recent weight gain, and previous thyroid cancer, thyroidectomy, and thymoma. All these conditions were considered in the differential diagnoses for her clinical depression. Current studies, however, indicate a potential correlation between depression and MG, which suggests that the presence of MG may contribute to the development of depression. To gain a better understanding of the link between MG and depression, further research is warranted. Future studies should explore the intricate relationship between these two conditions, specifically considering both the psychosocial factors and physiological aspects of MG that may contribute to depression in these patients. Such investigations will aid in the development of targeted interventions and effective management strategies for individuals with comorbid MG and depression, which will ultimately improve their overall well-being and QoL. 
